# A case report of Andersen-Tawil syndrome misdiagnosed with myodystrophy

**DOI:** 10.3389/fneur.2023.1170693

**Published:** 2023-06-30

**Authors:** Xiuqin Zhao, Hengbing Zu, Kai Yao

**Affiliations:** Department of Neurology, Jinshan Hospital Affiliated to Fudan University, Shanghai, China

**Keywords:** Andersen-Tawil syndrome, KCNJ2, malignant hyperthermia, anesthetic considerations, long-term exercise test

## Abstract

Andersen-Tawil syndrome (ATS) is a rare periodic paralysis caused by the KCNJ2 gene mutation. Here, we report on an ATS patient misdiagnosed with myodystrophy. A 66-year-old man presented with a 60-year history of episodic weakness in the proximal muscles of the upper and lower limbs. The man has been diagnosed with muscle pathology and has undergone genetic examinations in many hospitals since childhood. We conducted a correct diagnosis in combination with the patient’s history, electrical physiology, and genetic analysis and identified a heterozygous KCNJ2 gene variant (c.220A > G; p.T74A). Patients with ATS can develop permanent myasthenia characterized by chronic progressive myopathy. ATS patients should also pay special attention to the risks of anesthesia in surgery, including malignant hyperthermia (MH), muscle spasms affecting tracheal intubation or ventilation, and ventilator weakness. Early diagnosis and therapy could help delay the onset of myasthenia and prevent risks associated with anesthesia accidents.

## Introduction

ATS is a rare form of periodic paralysis and accounts for less than 10% of all periodic paralysis (1,500,000) (1, 2). This disease is caused by a KCNJ2 gene mutation, which encodes the inward-rectifying potassium channel known as kir2.1 (3, 4). The incorrect folding of the kir2.1 protein and the abnormal function of the channel results in a dominant negative effect on the potassium channel current, leading to decreased inward rectifying potassium current (5), impairing the repolarization process of the resting action potential of muscle fibers, affecting the excitability of skeletal and cardiac muscles, and subsequently causing periodic paralysis and arrhythmia (skeletal muscle and heart symptoms) (6). Subtle characteristic facial and skeletal abnormalities are often indicative signs used to diagnose ATS. The expression of Variant KCNJ2 leads to malfunctions in the potassium channel, affecting the spatial distribution Vmem (resting potential) and disrupting the normal pattern of membrane voltage potential regionalization, which, in turn, leads to misexpression of the craniofacial patterning genes during embryonal development in mice (7). Many studies have observed that the craniofacial features of ATS represent a spectrum of phenotypic manifestations, which include a broad forehead, low-set ears, broad nose, and maxillary and mandibular hypoplasia. Skeletal anomalies included short stature, small hands and feet, scoliosis, and clinodactyly of the fifth finger and toe (8). ATS patients also commonly exhibit symptoms such as periodic paralysis, cardiac abnormalities (including ventricular arrhythmia, prolonged Q-T interval, and inverted U wave), and myopathy (4, 9). To clinically diagnose ATS, the presence of at least two out of the following three cardinal features is typically required: periodic paralysis of skeletal muscles, characteristic dysmorphic features, or typical cardiac findings (6, 9).

## Case report

**Figure 1:**
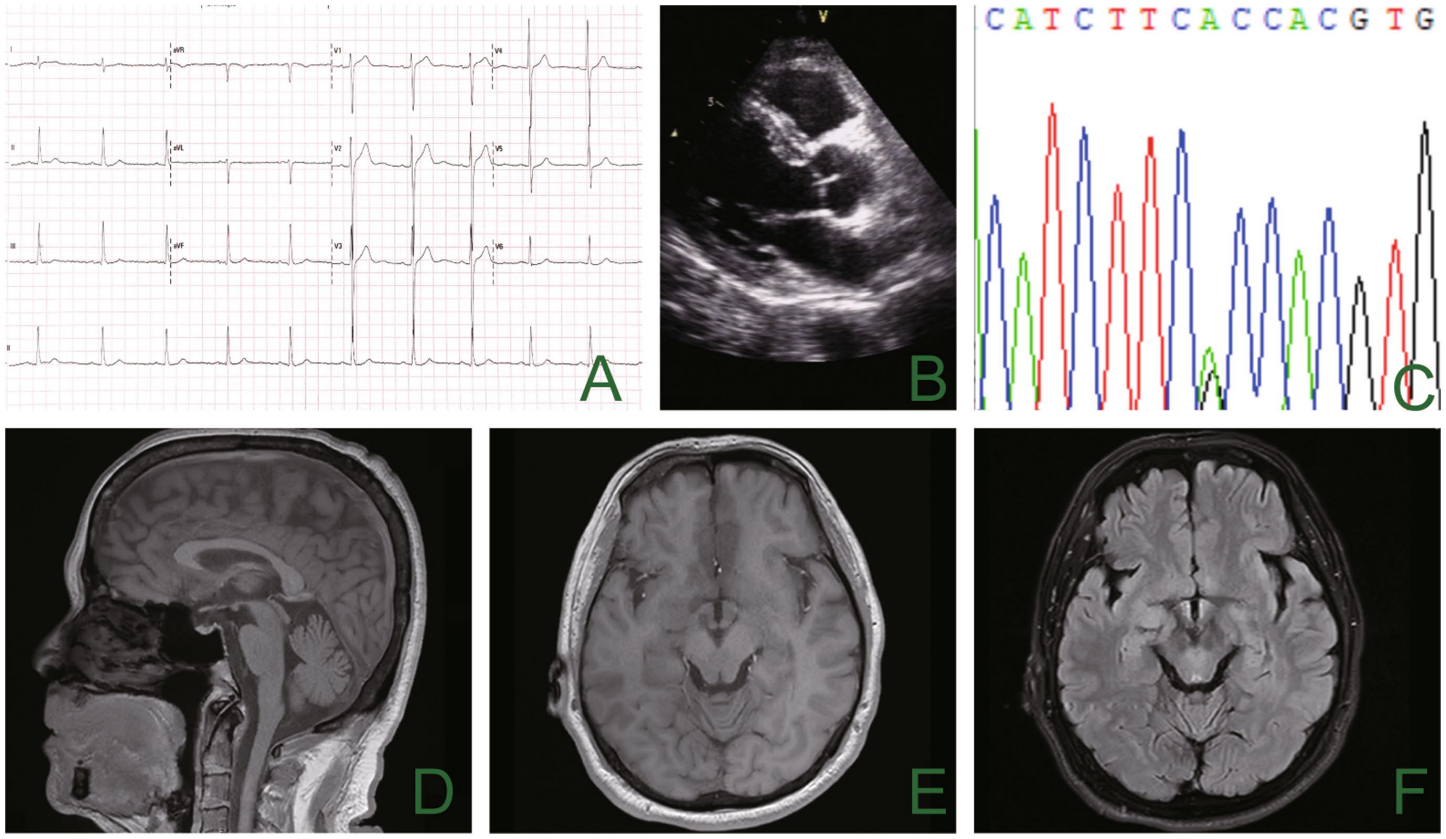
The electrocardiogram showed inverted U waves **(A)**, and the echocardiogram showed no apparent abnormality **(B)**. Genetic analysis using sequencing chromatograms identified a heterozygous KCNJ2 gene variant (c.220A>G; p.T74A) **(C)**. There was no noticeable abnormality in cranial magnetic resonance imaging **(D–F)**.

**Figure 2:**
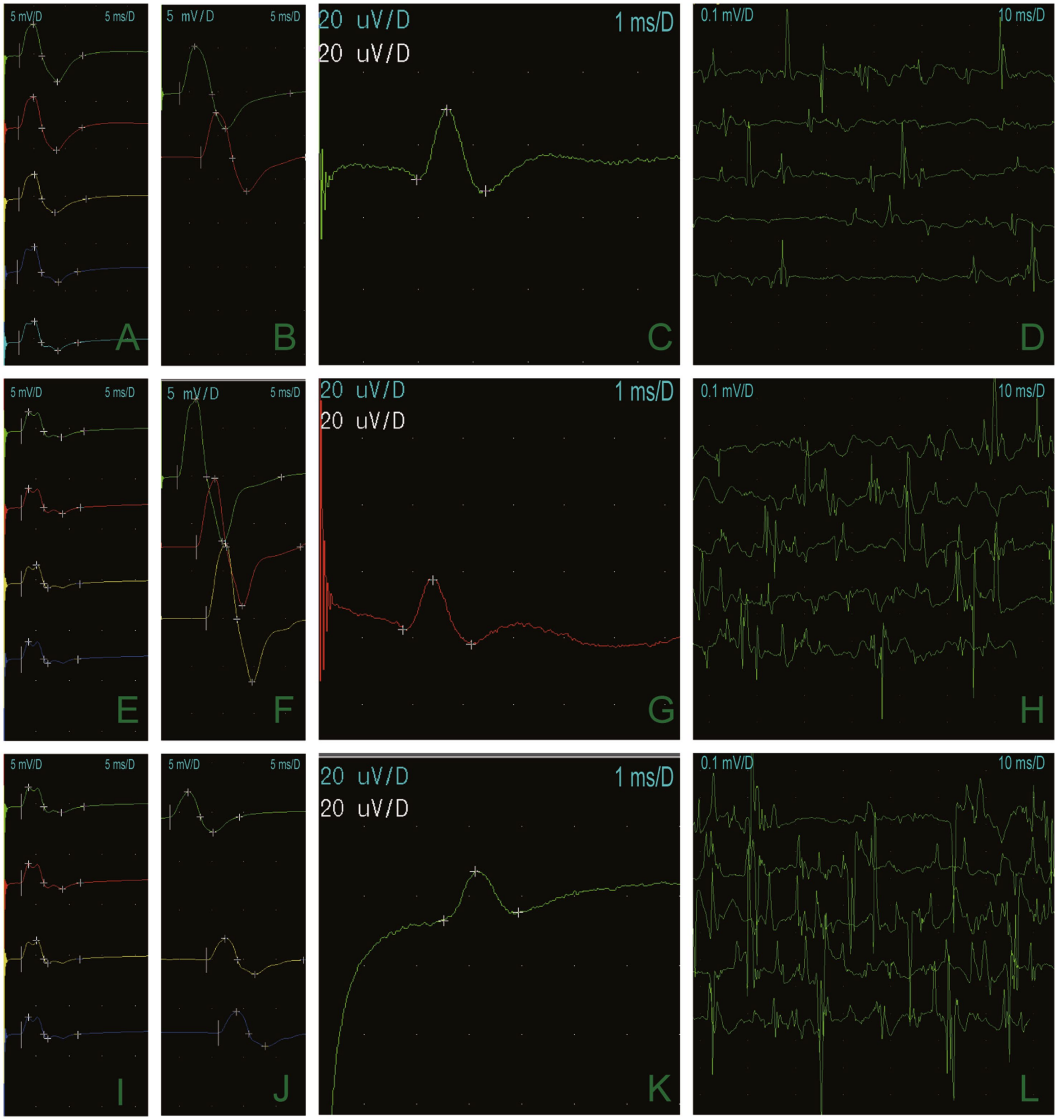
Long-term exercise test results demonstrated that the CMAP amplitude of the right little finger abductor muscle was 74.4% lower than it was at baseline, and the area was reduced by 66% **(A,E,I)**. The median nerve **(B)**, the ulnar nerve **(F)**, and the peroneal nerve **(J),** and motor conduction were normal. Sensory conduction of the median nerve **(C)**, the ulnar nerve **(G)**, and the superficial peroneal nerve **(K)** was normal. Electrophysiologic concentric needle examination fibrillation and a sharp positive wave at resting potentials **(D)**. Light contraction showed a narrow waveform **(H,L)**.

A 66-year-old man presented with episodic limb weakness beginning at 2 years of age, sometimes in both lower extremities and sometimes in both the upper and lower limbs. Prolonged walking was often the predisposing factor, with seizure frequency occurring once or twice a month. With age, he appeared to have permanent muscle weakness, manifested by difficulty in running, squatting, standing up, and lifting the upper limbs. During this period, he visited many hospitals to check his creatine kinase (CK) levels, which ranged from approximately 600–1,000 U/L. Muscle biopsies of his calf, shoulder, and back were performed at the ages of 13, 20, and 26 at two hospitals. The examination results showed no abnormalities, leading to a suspicion of muscular dystrophy as a possible cause. He underwent genetic testing at the age of 26, but the diagnosis was still inconclusive. Twenty-five years ago, an electrophysiologic concentric needle examination of the iliopsoas muscle, the gluteus maximus, and the lumbar paraspinal muscles indicated the presence of myopathy in the patient. There was a suspicion of limb-type muscle malnutrition, but treatment with coenzyme Q10 was found to be ineffective. It is worth noting that he had all his teeth extracted due to difficulties during intubation during vocal cord polyp surgery and was found quadriplegic after general anesthesia 10 years ago. The doctor conducted a cervical spine magnetic resonance imaging (MRI) on the patient, but the results showed no abnormalities. The patient complained of limb weakness, which progressively worsened in the morning before admission. He fell to the ground, could not stand up, and was brought to the emergency department of our hospital. Physical examination of the patient’s proximal part of the extremities presented amyotrophy (Medical Research Council Grade 4) and decreased tendon reflexes, and negative bilateral pathological signs. The electrocardiogram of our patient showed a U-wave inversion (Figure 1), and the 24-h dynamic electrocardiogram showed 245 premature ventricular contractions (PVCs) in a total of 91,847 heartbeats. The EMG examination revealed narrow and irregular waves during the needle examination, with no pronouncement of the fibrillation. A long-term exercise test indicated a significant decrease in the compound muscle action potential (CMAP) amplitude of the right little finger abductor muscle, measuring 74.4% lower than the baseline and a 66% reduction in the area (Figure 2).

**Figure 3:**
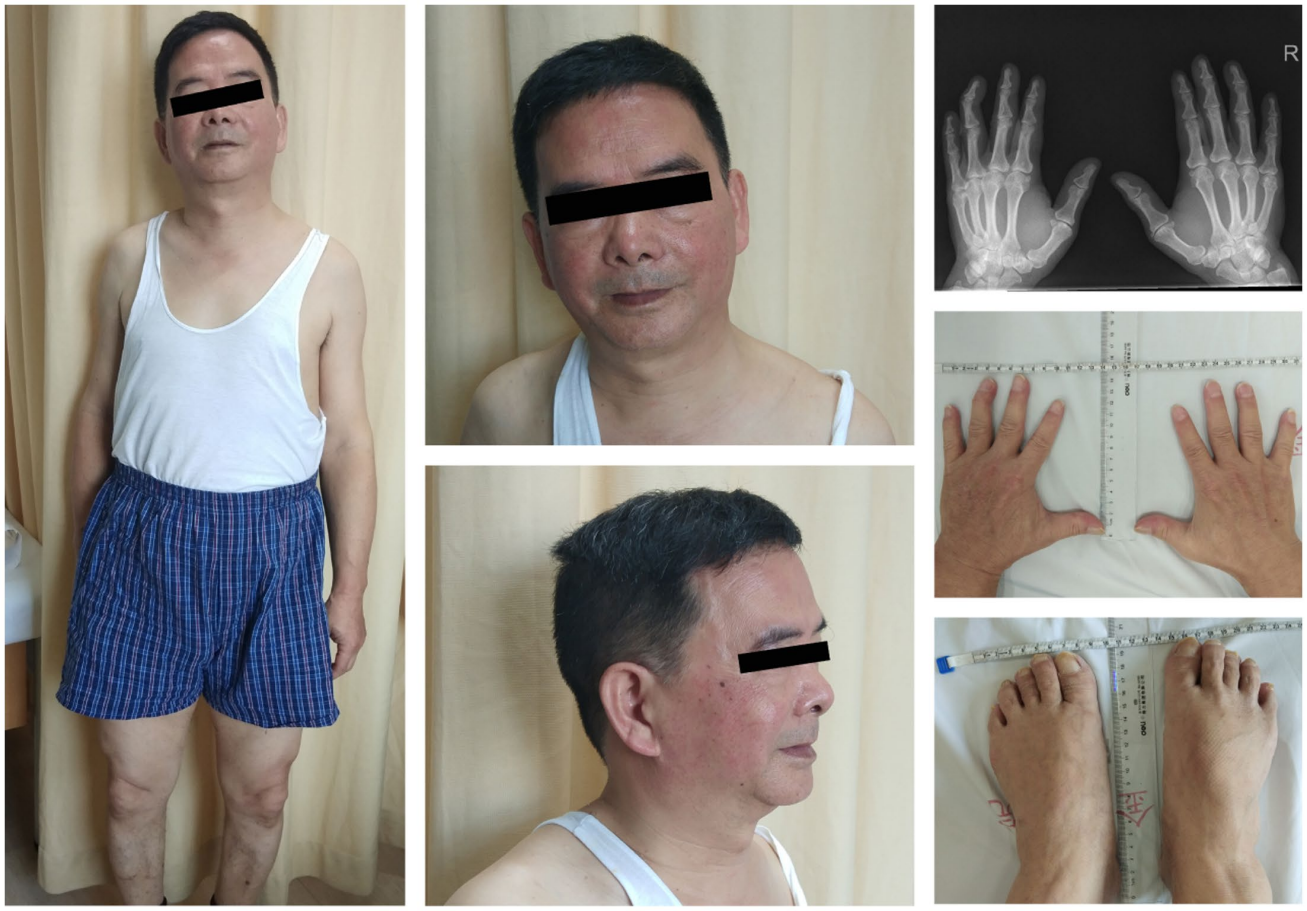
Short stature, facial features (AP and lateral), low-set ears, hypoplastic mandible, small hands and feet, clinodactyly of the fifth toe, and skeletal anomalies of the patient with ATS (permission was granted by the patient).

The blood test results showed a potassium level of 2.9 mmol/L (normal value: 3.5–5.5 mmol/L). Additionally, the CK (creatine kinase) level was measured at 1,600 U/L, lactic acid level at 2.8, and lactate dehydrogenase (LDH) at 224 U/L. We considered the possibility of ATS given the patient’s characteristic physical features, including short stature, small hands and feet, clinodactyly of the fifth toe, hypoplastic mandible, and low-set ears (Figure 3). An electrocardiogram revealed slightly inverted U waves. Further genetic analysis using the sequencing system (Amplicon Gene, Inc.) identified a heterozygous c.220A > G mutation in the exon 2 region of chromosome 17 of the KCNJ2 gene. This mutation resulted in the substitution of alanine with threonine at amino acid 74 (p.T74A) (Figure 1), which was reported to be a pathogenic mutation for ATS (10). Our patient had special physical characteristics. Based on the positive results of the long-term exercise test, muscle symptoms, and the genetic testing outcome, it was determined that he was not affected by the myopathic condition but rather ATS.

## Discussion

The misdiagnosis of ATS as a muscle disease has rarely been reported before. Muscular dystrophy or other muscle disorders are often characterized by muscle weakness, muscle atrophy, and increased CK, and electromyography showed myogenic damage. These similarities in symptoms and test results can often lead to confusion and misdiagnosis in cases similar to our patients. Periodic paralysis can cause episodic and permanent weakness caused by fibrotic and fatty replacement, and the incidence of permanent weakness is 68% (11). Studies have found that the biopsy of the left rectus femoris muscle revealed the characteristic vacuolar myopathy caused by the dilation of the T-tube, accompanied by occasional degeneration and regeneration of muscle fibers in periodic paralysis (12). A morphological follow-up of a muscle biopsy showed mild vacuolar changes at the age of 12. After 17 years, a subsequent examination showed that 15% of the muscle fibers exhibited tubular aggregates and medium-grade myopathy (13). The reports above describe the relationship between muscle pathology and permanent weakness. Our case showed permanent weakness at approximately 28 years, which is consistent with the above description.

Jeong et al. conducted a whole-body muscle MRI analysis on periodic paralysis patients and found that muscle fatty infiltration and atrophy are present in primary periodic paralysis, especially in older individuals, which are suggestive of chronic progressive myopathy (14). The whole-body muscle MRI demonstrates a selective pattern of muscle involvement affecting the posterior compartment of the lower leg and the anterior thigh muscles. Moreover, fatty muscle infiltration in these areas is more frequent and increases with age (15). Horga et al. reported that a man who presented with progressive proximal muscle weakness and mildly elevated CK was diagnosed with Becker muscular dystrophy (BMD) after a muscle biopsy showed abnormal myopathy in childhood. However, it was confirmed to be ATS by electromyography and genetic testing afterward (16). This case is similar to our case, where the patient also presented chronic and persistent limb muscle weakness, muscle atrophy, and a significant manifestation of myopathy in electromyography (EMG) examination, combined with previously normal blood K values and an abnormally increased blood CK value, which led to confusion with muscular dystrophy. The history of recovering muscle weakness during childhood could distinguish ATS from muscular dystrophy. Besides, ATS patients may have special appearance characteristics and experience a decrease in CMAP amplitude after a long exercise experiment. Permanent muscle weakness significantly impacts the patient’s quality of life, underscoring the importance of improving diagnostic approaches to reduce the time between the onset of the disease and the age of diagnosis.

As a special type of periodic paralysis, ATS patients with periodic paralysis are susceptible to various anesthesia-related complications, including malignant hyperthermia (MH), muscle spasms, tracheal intubation, and extubation failure (17–19). MH is a rare pharmacogenetic disorder of the skeletal muscle, triggered by sensitivity to volatile inhalation anesthetic gasses (e.g., sevoflurane, isoflurane, and so on) and depolarizing muscle relaxants (e.g., succinylcholine), which leads to a rise in core body temperature, tachycardia, hypermetabolism, pH value (pH) imbalance (hypoxemia, hypercapnia, and metabolic acidosis), liver and kidney damage, and hypermetabolism in the skeletal muscle (20, 21). It had an estimated mortality rate of 70–80% (22). Hypokalemic periodic paralysis was easy to trigger MH during general anesthesia (23–27). In addition, the use of depolarizing neuromuscular blocking drugs in ion channelopathies patients may lead to uncontrollable skeletal muscle hypermetabolism and sustained muscle contraction (28), such as generalized muscle spasms and masseter spasms, which can thus affect tracheal intubation or ventilation. In serious cases, it may cause respiratory muscle weakness and require prolonged mechanical ventilation (29–34). In an assessment of 109 patients with genetically confirmed skeletal muscle channelopathy, 17% (10 out of 59) reported worsening symptoms after general anesthesia independent of the duration of surgery, with high potassium periodic paralysis (29%) being the most common. A few patients experienced episodes of weakness after anesthesia, and nine of these patients also experienced prolonged recovery time after general anesthesia, independent of the duration of the procedure. There were no reports of laryngospasm during anesthesia (35).

In conclusion, it can be explained why our patient experienced difficulty with tracheal intubation in anesthesia surgery and paralysis when he woke up after surgery. According to the literature, patients with periodic anesthesia are susceptible to various anesthesia-related complications. Given the presence of craniofacial malformation and the potential cardiac risks associated with ATS, it is crucial to give significant attention to the possibility of difficult airways and an increased risk of severe arrhythmia during anesthesia (36). Therefore, the administration of neuromuscular blocking agents to ATS patients is a contentious issue. In patients with ATS or related family history, we believe that the use of strong inhalation anesthetics or suxamethonium should be used with utmost caution to prevent the occurrence of MH or other severe complications (37).

## Conclusion

The number of confirmed skeletal muscle channelopathy cases is increasing with the use of next-genetic sequencing, but its diagnosis remains challenging. We diagnosed an ATS patient who had been misdiagnosed with myopathy for a long time. Arrhythmias, typical craniofacial features, and a decrease in CMAP amplitude after a long exercise experiment are the key points for diagnosis. In addition, we described the anesthetic risks of ATS patients for the first time, including MH, muscle spasms, failure of tracheal intubation and extubation, and so on. More studies are needed to confirm how to diagnose ATS early, manage it, and prevent the risk of anesthesia accidents in the future.

## Data availability statement

The datasets presented in this study can be found in online repositories. The names of the repository/repositories and accession number(s) can be found in the article/supplementary material.

## Ethics statement

Written informed consent was obtained from the individual(s) for the publication of any potentially identifiable images or data included in this article.

## Author contributions

XZ enrolled the clinical data and wrote the draft. KY and HZ revised the manuscript and supported the project. All authors contributed to the article and approved the submitted version.

## Conflict of interest

The authors declare that the research was conducted in the absence of any commercial or financial relationships that could be construed as a potential conflict of interest.

## Publisher’s note

All claims expressed in this article are solely those of the authors and do not necessarily represent those of their affiliated organizations, or those of the publisher, the editors and the reviewers. Any product that may be evaluated in this article, or claim that may be made by its manufacturer, is not guaranteed or endorsed by the publisher.
